# Speech perception in adolescents with pre-lingual hearing impairment with cochlear implants

**DOI:** 10.1590/S1808-86942011000200003

**Published:** 2015-10-19

**Authors:** Izi Patricia Souza de Souza, Rubens de Brito, Ricardo Ferreira Bento, Maria Valéria S. Goffi Gomez, Robinson Koji Tsuji, Mariana Hausen-Pinna

**Affiliations:** 1 Specialist - Audiologist; 2 Associate Professor of Otorhinolaryngology - University of São Paulo Medical School - FMUSP; 3 Full Professor of Otorhinolaryngology - FMUSP; 4 PhD. Audiologist - Otology Group - FMUSP; 5 PhD. Assistant Physician - Otology Group - FMUSP; 6 MSc. Assistant Physician - Otology Group - FMUSP. Ambulatório de Implante Coclear do grupo de Otologia do Hospital das Clinicas da Faculdade de Medicina da USP

**Keywords:** cochlear implantation, adolescente health, deafness

## Abstract

Profound hearing loss is a disability that affects personality and when it involves teenagers before language acquisition, these bio-psychosocial conflicts can be exacerbated, requiring careful evaluation and choice of them for cochlear implant.

**Aim:**

To evaluate speech perception by adolescents with profound hearing loss, users of cochlear Implants.

**Study Design:**

Prospective.

**Materials and Methods:**

Twenty-five individuals with severe or profound pre-lingual hearing loss who underwent cochlear implantation during adolescence, between 10 to 17 years and 11 months, who went through speech perception tests before the implant and 2 years after device activation. For comparison and analysis we used the results from tests of four choice, recognition of vowels and recognition of sentences in a closed setting and the open environment.

**Results:**

The average percentage of correct answers in the four choice test before the implant was 46.9% and after 24 months of device use, this value went up to 86.1% in the vowels recognition test, the average difference was 45.13% to 83.13% and the sentences recognition test together in closed and open settings was 19.3% to 60.6% and 1.08% to 20.47% respectively.

**Conclusion:**

All patients, although with mixed results, achieved statistical improvement in all speech tests that were employed.

## INTRODUCTION

Cochlear implants are the treatment of choice for patients with severe to profound sensorineural hearing loss which does not respond to conventional hearing aids^1^. Children with prelingual hearing loss, being congenital or not, and who received a cochlear implant in their first year of life had significant gains insofar as hearing development is concerned, including speech perception; however, with longer times of hearing deprivation, lower are the speech perception rates after the implant, and greater are the difficulties in speech development^2^.

When treating teenagers with prelingual hearing loss, the long time of hearing deprivation reduces considerably the opportunity of gaining speech recognition after surgery. Therefore, when indicating cochlear implant to a heterogeneous group in terms of etiology, hearing impairment duration, cognition and language, we must take into account the parents’ expectations, family dynamics, the patient's wishes, his relationship with his social group, what are the implications in his identity - such as the patient's degree of maturity and affective-emotional conditions^3^, ^4^. In the recent past, cochlear implants were contraindicated in teenage patients with prelingual hearing loss, and it still is a very controversial matter, a procedure which is carried out in just a handful of public hospitals.

Our opinion has changed with the progress achieved in speech processors, which are now able to provide some degree of speech recognition in this population. Nonetheless, it is necessary to review our concepts of benefit and good results, thus restructuring the pre-implant evaluation which started to emphasize language assessment, social insertion and expectations concerning the cochlear implant, besides enjoying hearing with a hearing aid^5^, ^6^.

The few studies about the benefits brought about by the cochlear implant to teenage patients have varied results^7^, ^8^, ^9^, ^10^. This can be explained by the group's heterogeneity and the types of implants used^11^. Moreover, the small sample of the studies makes it difficult to carry out a proper statistical analysis. Having said that, our goal with the present study was to assess speech perception in adolescents with severe to profound bilateral, sensorineural and prelingual hearing loss with cochlear implants.

## SERIES

### Participants

This study was carried out in the Cochlear Implant Ward of the Otology Group of the University of São Paulo Medical School, and it was approved by the Ethics in Research Committee of the institution, under protocol # 1061/08.

25 adolescents were included with the following criteria:

- Age between 10 and 17 years and 11 months
- Prelingual hearing loss
- Severe to profound sensorineural hearing loss
- All the patients used hearing AIDS before the cochlear implant.

The assessment was carried out individually with each patient, before the cochlear implant and 2 years after they started using it, and during the two year os CI use, the patients were followed by an audiologist for the practice and development of hearing skills.

Only 2 of the 25 patients could not be assessed after 2 years of using the CI, because they did not come to the appointment. Thus, in the result analysis we included 23 adolescents.

Fifteen of the participants were females and 8 were males (Table 1).

**Table 1:** Demographic data from the 23 patients in the study.

Patient	Gender	Etiology	Hearing aid use time	Implant ear	Age upon activation	Implant	500-1000-2000Hz with CI hearing thresholds
1	Female	Congenital	3 years	Right	10.4	Nucleus 24 M/K	30-15-15 dB
2	Female	Congenital	9 years	Right	10.4	Nucleus 24 M/K	40-35-35 dB
3	Female	Meningitis	10 years	Right	11	Nucleus 24 M/K	30-30-25 dB
4	Male	Meningitis	9 years	Right	10	Nucleus 24 M/K	45-40-45 dB
5	Female	Congenital	6 years	Right	11.4	Nucleus 24 M/K	40-35-35 dB
6	Female	Congenital	5 years	Right	10	Freedom SP	25-30-20 dB
7	Male	Congenital	8 years	Right	10.4	Medel COMB40+	45-40-40 dB
8	Female	Congenital	10 years	Right	12.3	Nucleus 24 M/K	30-20-30 dB
9	Male	Congenital	9 years	Right	12.1	Nucleus 22	25-15-25 dB
10	Male	Congenital	2 years	Right	12.4	Medel COMB40+	30-15-25 dB
11	Female	Congenital	7 years	Left	10.4	Nucleus 24 M/K	30-15-25 dB
12	Female	Congenital	11 years	Right	12.8	Nucleus Freedom	35-35-35 dB
13	Female	Congenital	8 years	Right	10.4	Clarion	40-40-40 dB
14	Female	Congenital	9 years	Right	13.9	Nucleus 24 M/K	35-35-40 dB
15	Female	Congenital	13 years	Left	14.1	Nucleus Freedom	40-30-30 dB
16	Male	Congenital	12 years	Left	14.8	Clarion	30-20-20 dB
17	Female	Congenital	13 years	Left	14.8	Nucleus 24 M/K	30-25-25 dB
18	Female	Meningitis	14 years	Right	15.6	Nucleus 24 M/K	55-40-40 dB
19	Male	Congenital	14 years	Left	15.2	Nucleus 22	30-25-25 dB
20	Male	Meningitis	16 years	Right	15.1	Nucleus 24 M/K	35-25-30 dB
21	Female	Rubella	16 years	Right	17.9	Nucleus Freedom	30-25-30 dB
22	Male	Meningitis	16 years	Left	17.9	Esprit 3G	15-30-25 dB
23	Female	Congenital	16 years	Right	17.9	Freedom SP	35-25-30 dB

## METHOD

Routinely, as part of patient selection, in order assess performance and to obtain the data necessary for programming, all implanted patients were submitted to speech perception tests before being submitted to cochlear implant and six months after the activation of the device. This test is a prospective quantitative analysis in which the selected patients were submitted to speech perception tests before and 6, 12 and 24 months after the cochlear implant device activation. For this study, we considered the results obtained in the tests after 24 months of use in order to compare it with the pre-implant results.

The speech perception test was carried out live and the complete evaluation protocol was described by Gómez et al.^12^. The results from the following tests were used (ascending order of difficulty): *four choice*, vowel recognition, closed set sentences recognition, open set sentence recognition, in the audio-visual and auditory modes. All the tests had a maximum score of 100% and minimum of 0%, and each correct answer or wrong answer corresponds to a percentage which varies according to the total number of sentences or words existing in the test; for instance, the closed set sentences recognition test is made up of 10 sentences, thus, each one corresponds to a total of 10%.

The statistical analysis was carried out by the *Statistical Package for Social Sciences* (SPSS) software, version 16.0 for Windows (SPSS Inc, Chicago - IL). We analyzed the correlation between the speech recognition tests through the use of the Pearson's and Spearman's correlation coefficients. The pre and post cochlear implant scores comparison from the speech recognition tests were carried out using the non-parametric Wilcoxon test for paired samples. To do that, we considered the statistically significant differences, having *p* values below 0.05.

## RESULTS

**Figure 1:**
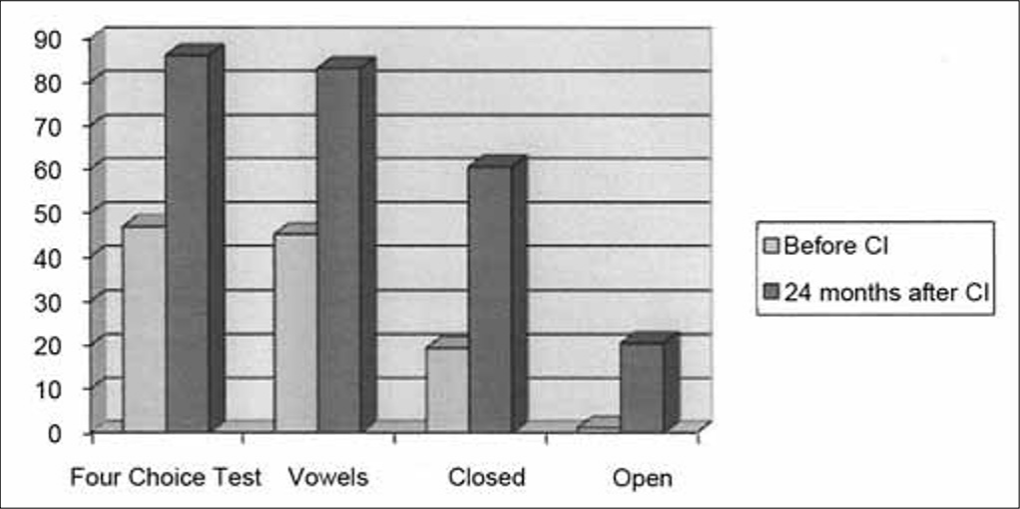
Mean value of the percentage of correct answers for the *four choice*, vowels, closed and open sentences before and 24 months after the cochlear implant.

All the patients were submitted to speech perception tests before the cochlear implant and 2 years after it. We compared the results of the tests employed before and 24 months after cochlear implant use. The mean value of correct answers in the *four choice* test before and after the cochlear implant was of 46.9% and after 24 months of device use, this mean value went up to 86.1%. In the vowel recognition test, the mean values were between 45.13% to 83.13%. in the closed set sentences recognition test, the mean value before the cochlear implant was 19.3% and after 2 years, the mean was 60.6%. In the open set sentences recognition test, the mean percentage of correct answers before the implant was 1.08% and after 2 years, this value went up to 20.47%. (Table 2) (Fig. 1).

**Table 2:** Results from the speech recognition in percentages of correct answers.

Patient	Four Choice Before CI	Four Choice After CI	Vowels Before CI	Vowels after CI	Closed sentences before CI	Closed sentences after CI	Open sentences before CI	Open sentences after CI
1	50%	50%	20%	100%	0%	0%	0%	0%
2	33%	92%	46,60%	100,00%	0%	90%	0%	0%
3	41%	83%	0%	73%	0%	90%	0%	0%
4	0%	91%	0%	46%	0%	30%	0%	0%
5	0%	100%	0%	100%	0%	0%	0%	0%
6	16,60%	83%	26,60%	53,30%	0%	60%	0%	0%
7	66%	83%	0%	80%	0%	0%	0%	0%
8	0%	100%	40%	80%	0%	0%	0%	0%
9	75%	100%	90%	86%	10%	100%	0%	0%
10	90%	100%	66%	100%	50%	100%	0%	80%
11	0%	100%	40%	100%	0%	90%	0%	0%
12	0%	80%	73%	100%	50%	50%	0%	0%
13	58%	83%	46%	40%	0%	10%	0%	0%
14	0%	36%	0%	0%	0%	0%	0%	0%
15	83,30%	100%	73%	100%	0%	100%	0%	36%
16	16,60%	50%	13,30%	80%	0%	0%	0%	0%
17	100%	100%	80%	100%	90%	100%	0%	90%
18	41,60%	50%	100%	100%	50%	100%	25%	25%
19	66%	100%	53%	73,30%	35%	75%	0%	0%
20	100%	100%	53%	100%	50%	100%	0%	50%
21	100%	100%	100%	100%	80%	100%	0%	70%
22	92%	100%	47%	100%	30%	100%	0%	70%
23	50%	100%	70%	100%	0%	100%	0%	50%

## DISCUSSION

Adolescence is a very particular stage of development. The psychological changes which happen at this time, together with the body changes, cause numerous bio-psycho-social conflicts in the individual; thus, it is necessary to be cautious in the assessment and selection of candidates to cochlear implant, especially in individuals with prelingual hearing loss, in whom hearing results are very heterogeneous.

The main goal of cochlear implants in adolescents with prelingual hearing loss is to audiologically enable them to perceive and recognize speech, and the hearing thresholds in all the individuals after the cochlear implant were enough to have access to it.

There is a consensus in assessing the results from the speech perception tests 12 months after continuous use of the cochlear implant, when there would already be a learning curve flattening 12, 13. In the present study, we chose to assess our patients 24 months after, because it was a heterogeneous group and with a long time of hearing deprivation, thus, with a particular and slow learning time^14^.

We could also observe this, because despite the individuals being adolescents with prelingual hearing loss, the results vary among themselves - which can be justified by another study between the differences in speech recognition performance differences considering characteristics such as: time to diagnosis, etiology, onset of hearing aid use, speech therapy strategy and emotional characteristics^15^, ^16^.

Regardless of the results obtained in the four choice, vowel recognition and closed and open set sentence recognition, all individuals reported increases in selfconfidence and improvement in the general well-being after they started using the cochlear implant. This fact is associated with the idea of improving the quality of life of these individuals, and being teenagers, this represents a very important issue in the psycho-social aspect of their lives. Psychological status is also a determining factor for satisfactory results or not in the speech perception tests of adolescents^17^, ^18^. We can use this study as a response to the short progress of patient # 14, since he was going through a critical period of depression and did not effectively use the implant for some months.

## CONCLUSION

Based on the analysis of the results from the tests made with the 23 adolescents, we have concluded that:

• All the patients obtained sufficient hearing thresholds in order to have Access to speech sounds;
• All the patients, although with heterogeneous results, obtained statistical improvements in all the speech tests employed.
